# Taxonomy of chronic illness research recruitment: a restricted scoping review

**DOI:** 10.1186/s12913-025-13115-8

**Published:** 2025-07-29

**Authors:** Rosalynn C. Austin, Bjørg Karlsen, Alison Richardson, Glyn Elwyn, Marianne Storm, Anne M. L. Husebø, Kristin H. Urstad

**Affiliations:** 1 Department of Public Health, Faculty of Health Sciences, University of Stavanger, Stavanger, 4036 Norway; 2 Department of Cardiology, Portsmouth Hospitals University NHS Trust, Portsmouth, PO6 3LY UK; 3 National Institute of Health and Care Research (NIHR) Applied Research Collaborative (ARC) Wessex, Southampton, SO17 1BJ UK; 4 University Hospital Southampton NHS Foundation Trust, Southampton General Hospital, Mailpoint 11, Clinical Academic Facility (Room AA102), South Academic Block, Tremona Road, Southampton, SO16 6YD UK; 5 School of Health Sciences, Building 67 University of Southampton, University Rd, Southampton, SO17 1BJ UK; 6 The Dartmouth Institute for Health Policy and Clinical Practice at the Geisel School of Medicine at Dartmouth College, Lebanon, NH USA; 7 Research Group of Nursing and Health Sciences, Research Department, Stavanger University Hospital, Stavanger, Norway; 8 Faculty of Health Sciences and Social Care, Molde University College, Molde, Norway

**Keywords:** Research trials, Recruitment, Restricted review, Facilitators and barriers, Qualitative synthesis, Mixed methods, Taxonomy

## Abstract

**Background:**

Chronic illness prevalence is increasing and research recruitment in these populations remains challenging. Individuals with chronic illness often have poorer quality of life, restricted access to hospitals where research occurs, and can be reluctant to participate. Researchers need multiple simultaneous strategies to achieve success. No taxonomy of recruitment factors in chronic illness research could be identified in the literature. This paper aims to describe a comprehensive taxonomy of recruitment for chronic illness research (inclusive of a nursing focus) to inform the design and reporting of recruitment strategies by creating a list of practical questions.

**Methods:**

A restricted scoping review was conducted on articles reporting on recruitment factors in chronic illness research. Main search restrictions were the number of years and databases searched with broad eligibility criteria. Included articles were critically assessed and data extracted. A code book was used to examine findings and results sections line by line, both deductively and inductively. The final codebook and the content of the codes informed the taxonomy construction and the practical questions.

**Results:**

Core components of research recruitment were identified as people, place, and project. The component of People included factors of researchers, clinicians, recruiters, and participants roles. The component of Place included factors of national or local research oversight institutions, healthcare environments, and community spaces. Finally, the component of Project included factors of research design, participant research journey, and research promotion. The final taxonomy informed a practical list of questions to aid researchers in the design and reporting of research recruitment strategies.

**Conclusions:**

The chronic illness research recruitment taxonomy describes and characterises factors reported to impact on research recruitment. It provides a framework for designing and reporting on recruitment strategies. While the taxonomy requires further testing, it is the first to offer a broad characterisation of recruitment factors in chronic illness research.

**Supplementary Information:**

The online version contains supplementary material available at 10.1186/s12913-025-13115-8.

## Background

Chronic illnesses are growing in prevalence [1, 2] and research recruitment in these populations has challenges related to restricted healthcare access for those with chronic illness [3]. Chronic illness is defined as any non-communicable illness with lasting impact on an individual’s health requiring treatment and management (major types include but are not limited to cardiovascular and respiratory diseases, cancers, and diabetes) [4]. Subpopulations common in chronic illness (e.g., older adults, women, and minority ethnic groups) are less well represented in research, making research less generalisable [5, 6]. Proactive steps need to be taken to ensure that research recruitment is considered from an inclusive and holistic approach.

Recruitment to research trials is often time-consuming and difficult [7–9]. Recruitment that is neither inclusive nor sufficient has implications for the generalisability of research [10, 11]. A previous literature review reported that despite multiple research studies that have investigated recruitment issues, challenges remain persistent [12]. Searches on research recruitment return large numbers of articles. Articles reporting on recruitment could be generally classified as either purposeful research investigations into recruitment (recruitment research), reflective case studies, or embedded recruitment reports. Existing literature reviews on recruitment challenges focused on the patient/participant [12–17], clinician [12, 18, 19], or researcher [12, 14–17, 19] experiences. Similarly, existing frameworks on recruitment strategies are either specific to maternity research [20, 21], focused on patient experience [22], or research design and delivery [23]. Recruitment tools created offer real-time evaluation of recruitment challenges [10, 24, 25] or assess the applicability and recruitment of a trial related to the design of a trial [26]. However, the existing literature on research recruitment has a singular focus (illness type, research design, personal characteristics, etc.). It follows that research recruitment in chronic illness depends on the perspectives of participants and clinicians. Additionally, the design and delivery of research study impact recruitment together with the interplay of all these components of research recruitment. A practical and holistic taxonomy of research recruitment in chronic illness was not identified.

The purpose of this review is to examine research recruitment literature across multiple chronic illness types, study designs, and perspectives to create a chronic illness research recruitment taxonomy, inclusive of a nursing focus.

Research question: What are the factors that impact recruitment of people with chronic illness into research?

## Methods

The scoping review framework of Arksey and O'Malley [27] was adapted by incorporating some principles of the restricted systematic review approach, as proposed by Plüddemann, Aronson [28]. Restrictions were applied, as it enabled the researchers to restrict the amount search returns based on available researcher time and resources while maintaining the core review elements (literature search, study selection, data extraction, critical assessment, synthesis, and publication) [28]. Restrictions for this review included search strategy characteristics, number of databases and years searched, and the amount of blinded article screening.

Search strategy was developed in partnership (EHM and RA) [29] and run in Embase, Ovid Medline, and PsycINFO on 02/08/23. Initial searches returned large numbers of search returns, so the search strategy excluded abstract content, and a nursing focus was added, as frequently nurses perform the work of recruitment. The same search was rerun on 12/02/24 to update the search pre-publication. Any literature reviews identified had the references hand searched for other possible articles to consider for inclusion.

Eligibility criteria are listed according to the SPIDER tool [30] in Table 1. As the aim of this scoping review was to identify any research recruitment related activity that may impact research recruitment and to ensure that the nursing perspective was captured multiple research study types were included (trials, cohort, etc.). A broader focus to identify research recruitment related activity reported influence recruitment should extend findings past the typically reported screening data typically found in research trials.

**Table 1:** Eligibility criteria for the review

	Inclusion	Exclusion
Sample	Any size, any chronic illness, adults (e.g., clinicians, researchers, patients who were invited to report on recruitment experiences)	Studies where participants were children, healthy volunteers, pregnant women, or chronic illnesses with altered cognitive abilities
Phenomenon	Reported impactful activities related to research recruitment in chronic illness research	
Design	Published peer reviewed articles of any research methodology published within the past 5 years	No available published data
Evaluation	Factors identified to either improve and/or limit recruitment to research trials/studies	
Research type	Qualitative, quantitative, and mixed methods	Protocol papers, abstracts, literature reviews, editorials, letters, etc

Heterogeneity: was expected due to the broad eligibility criteria of research methodology and participant types. A summarised description of the article characteristics of the articles was conducted. In this article the term *people* is used to represent any participant in an included articles (i.e., people with chronic illness, researchers, clinicians, administrators, and industry partners) who had contributed to the data on the recruitment factors and referred to as *people* in this article. A meta-synthesis was conducted by using the text within the results/findings sections (including figures, tables, and supplemental material).

Quality Assessment: was conducted using the Mixed Methods Appraisal Tool (MMAT) [31]. Included articles were assessed and rated as moderate or high quality based on the number of “yes” answers to MMAT questions. Articles were rated as high quality if only one question was answered as “no” or “can’t tell”. Following coding, the method used to evaluate if the quality of an article impacted on the coding used automatic counts in Nvivo [32]. The frequency of coding in articles and coding content, grouped by MMAT scores, was examined and compared [33].

Data extraction strategy: Data extraction of article details (e.g., participants, location, etc.) was organised in MS Excel [34] by RCA. All text within the results or findings sections (including figures, tables, and supplemental material) was extracted from the full article’s and organised in NVivo [32]. Inclusion of text from discussion section was made based on case-by-case evaluation dependent on article type. This was limited to reflective case study articles with a writing style merged results and discussion as the main body of the article.

Data synthesis and presentation: Thomas & Harden [35] framework for synthesis was used and adapted to facilitate the reporting of qualitative results. Components of recruitment (e.g., patients, clinicians, and research project design (Supplementary Material 1)) were used to build an initial codebook by the research group. RCA performed inductive line-by-line coding of extracted data to identify factors that impacted recruitment. During characterisation an abductive analysis [36] approach of iterative exploration meant a deductive re-coding occurred as inductive codes were re-organised into the code book. All new observations lead to the expansion and refinement of the initial code book. The original core components of patients, clinicians, and research project design were reorganised in this process to create the iteratively formed final components of people, place, and project. Finally, an iterative process informed the construction of the taxonomy which was founded on the final codebook (Supplementary Material 1). Practical questions were created on the foundation of the taxonomy by considering the content within each component and factor in an iterative discussion with all authors until agreement was reached.

Quantitative data was synthesised through descriptive analysis. Due to the expected heterogeneity and the focus of this work on qualitative data was anticipated to be limited to article characteristics (methodology, global location, number and type of participants.

## Results and discussion

### Review statistics

**Fig. 1:**
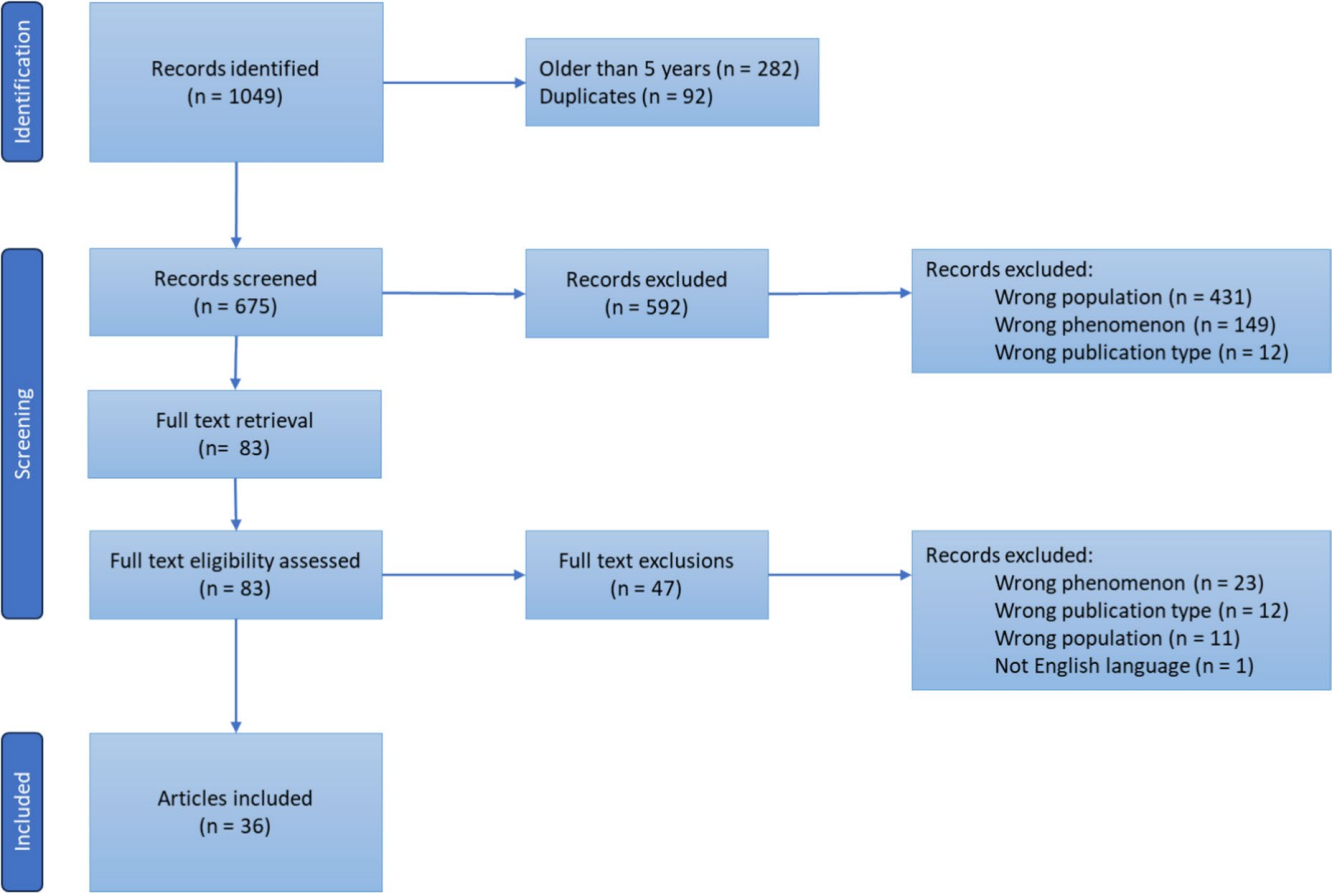
PRISMA flow diagram

The combined searches returned n = 1049 articles of which 36 met eligibility criteria. A sample (*n* = 300) of the search results were reviewed by a second blinded researcher (KHU, MALH, MS reviewed 100 titles/abstracts each). Conflicts (*n* = 4) were resolved by discussion. The PRIMSA flow diagram [37] demonstrates the detailed screening (Fig. 1).

The focus of these papers was on recruitment factors (e.g., facilitators or barriers) and articles were classified as: recruitment research: independent study which explored facilitators or barriers of recruitment, embedded recruitment analysis: within a larger research study/trial, and case study recruitment: reflexive exploration of recruitment strategies observed impact. Table 2 lists all included articles.

**Table 2:** List of included articles (grouped by methodology classification as defined in this article)

Article classification: Research recruitment						
Authors	Title (abbreviated)	Year	Journal (abbreviation)	Condition	Methodology	Location
Anzuoni, et al. [38]	Recruitment Challenges for Low-Risk…	2020	J Am Geriatr Soc	Older adults	Mixed Methods	USA
Bailey, et al. [39]	Staff and participant perceptions of…	2021	Cancer Causes Control	Cardiovascular	Qualitative	JAM
Bell, et al. [40]	Gatekeeping in cancer clinical trials in…	2020	Cancer Med	Cancer	Qualitative	CDN
Brehaut, et al. [41]	Using behavioral theory and shared…	2021	Trials	Cardiovascular	Qualitative	CDN
Coyle, et al. [42]	A secondary qualitative analysis of…	2022	Trials	Multiple	Qualitative	SCT
Crocker, et al. [43]	Recruitment and retention of…	2020	BJS Open	Chronic Pain	Quantitative	ENG
Duckham, et al. [44]	Strategies and challenges associated with…	2018	BMC Med Res Methodol	Older adults	Quantitative	AUS
Isaksson, et al. [45]	Identifying important barriers to…	2019	Trials	Neurological	Quantitative	SWE
Keruakous, et al. [46]	Research staff perspectives on cancer…	2021	Cureus	Cancer	Qualitative	USA
Laaksonen, et al. [47]	Success and failure factors of patient…	2022	Trials	Mixed	Qualitative	FIN
Legor, et al. [48]	Clinical research nurses'perceptions of…	2023	Contemp Clin Trials	Cancer	Qualitative	USA
McDermott, et al. [49]	Maximising recruitment to a randomised…	2021	Trials	Respiratory	Qualitative	ENG
Prout, et al. [50]	Maximising recruitment of research…	2022	Trials	Cancer	Qualitative	CYM
Realpe, et al. [51]	Barriers to recruitment to an…	2021	Bone Jt Open	Arthritis	Qualitative	ENG
Schmidt, et al. [52]	Improving Iowa research network patient…	2021	J Prim Care Community Health	Mixed	Quantitative	USA
Stafford, et al. [53]	Why did we fail? Challenges recruiting…	2019	Psycho-Oncology	Cancer	Qualitative	AUS
Vluggen, et al. [54]	Exploring factors influencing recruitment…	2020	Clinical Trials	Diabetes	Mixed Methods	NLD
Wharton-Smith, et al. [55]	Optimising recruitment to a late-phase…	2021	Trials	Respiratory	Qualitative	UZB

Article classification: Case Study						
Authors	Title (abbreviated)	Year	Journal (abbreviation)	Condition	Methodology	Location
Hall, et al. [56]	Recruitment of patients with de novo…	2018	Trials	Neurological	Qualitative	USA
Hays, et al. [57]	Recruitment issues in emerging adult…	2020	Nursing reports	Cardiovascular	Qualitative	USA
Imran, et al. [58]	Clinical research nurses, perspectives on…	2022	J Res Nurs	Respiratory	Qualitative	ENG
Koirala, et al. [59]	Conducting nursing research in low- and…	2020	Nurse res	Cardiovascular	Qualitative	NPL
Magwood, et al. [60]	High tech and high touch: Recruitment…	2021	Contemp Clin Trials Commun	Neurological	Qualitative	USA
Nichols, et al. [61]	Where have they gone…	2021	Online J Rural Nurs Health Care	Older adults	Qualitative	USA
Shropshire, et al. [62]	Barriers and Insights in participant…	2020	Nurs Sci Q	Chronic Pain	Qualitative	USA
Sullivan, et al. [63]	Castrate-resistant prostate cancer…	2018	Int J Palliat Nurs	Cancer	Qualitative	AUS
Taani, et al. [64]	Lessons learned for recruitment and…	2020	Contemp Clin Trials Commun	Cardiovascular	Qualitative	USA

Article classification: Embedded recruitment analysis						
Authors	Title (abbreviated)	Year	Journal (abbreviation)	Condition	Methodology	Location
Brickey, et al. [65]	Barriers to recruitment into emergency…	2022	BMC Palliat Care	Older adults	Quantitative	USA
Conefrey, et al. [66]	Strategies to Improve Recruitment to a…	2020	Clin Oncol	Cancer	Qualitative	ENG
Edwards, et al. [67]	Evaluating recruitment methods of…	2019	Int J Pharm Pract	Cancer	Quantitative	ENG
Johnson, et al. [68]	Hospital recruitment for a pragmatic…	2018	Trials	Neurological	Mixed Methods	USA
Lucas, et al. [69]	Recruiting endometrial cancer survivors…	2018	J Cancer Educ	Cancer	Quantitative	USA
Price, et al. [70]	Challenges of recruiting emergency…	2020	BMC Med Res Methodol	Chronic Pain	Qualitative	CYM
Stuckenschneider, et al. [71]	Recruiting patients for falls prevention…	2023	BMC Geriatrics	Older adults	Quantitative	DEU
Tew, et al. [72]	Site-specific factors associated with…	2023	Trials	Diabetes	Quantitative	AUS
Whelan, et al. [73]	Recruiting patients to a digital…	2021	Digital health	Respiratory	Quantitative	ENG

*USA* United States of America, *JAM* Jamica, *CDN* Canada, *SCT* Scotland, *ENG* England, *AUS* Australia, *SWE* Sweden, *FIN* Finland, *CYM* Wales, *NLD* Netherlands, *UZB* Uzbekistan, *NPL* Nepal, *DEU* Germany

^*^Standard 3 country abbreviations were used

**Fig. 2:**
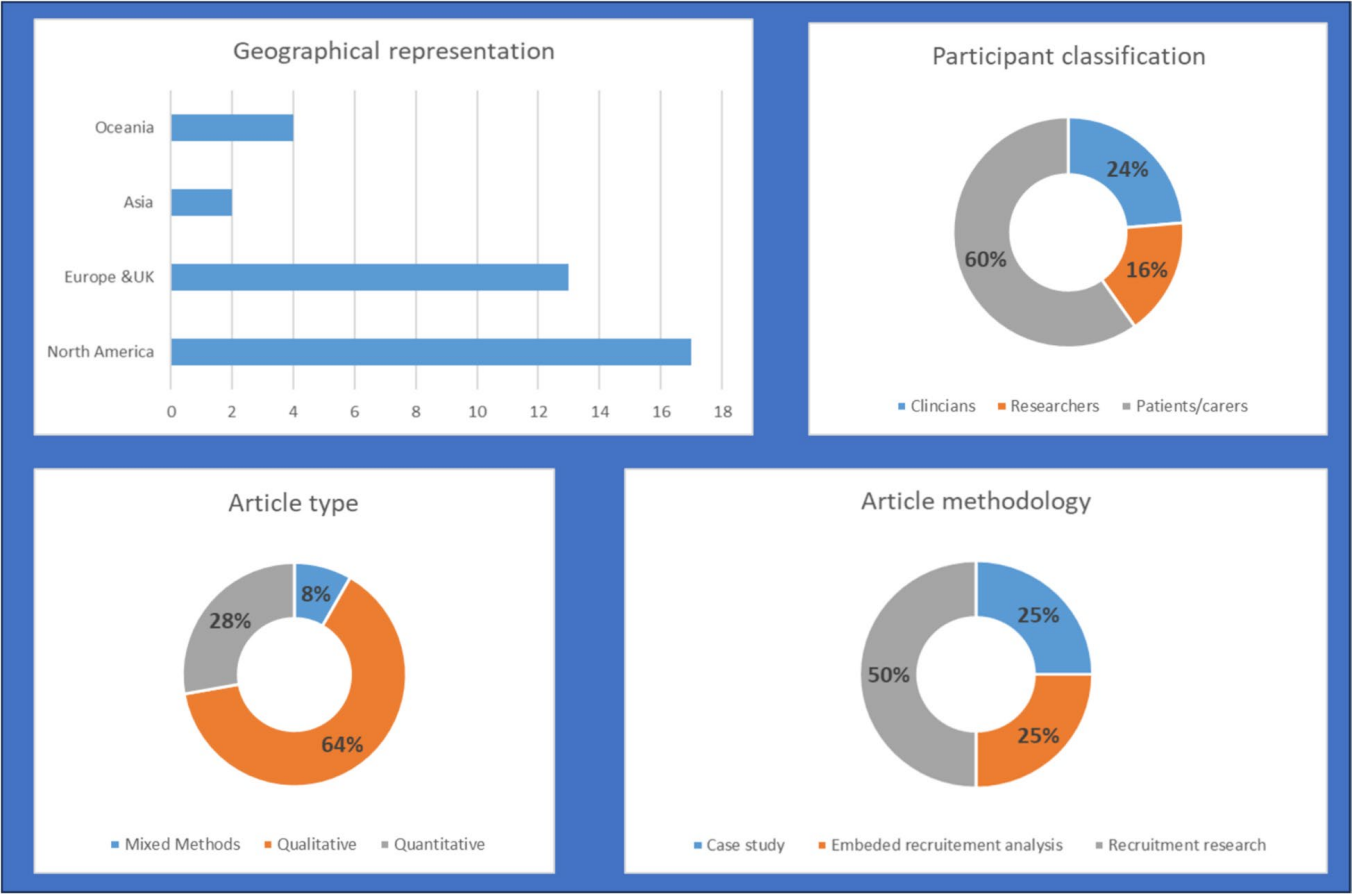
Description of included articles—global representation, participant classification, and article type and methodology

Included articles were from multiple countries and included the recruitment experiences of patients, clinicians, and researchers (Fig. 2).

Included articles reported on the experience of recruitment across multiple study types and trials (feasibility study *n* = 1, cohort studies *n* = 8, pilot trials *n* = 4, and clinical trials *n* = 23).

### Quality assessment

Based on MMAT [31] responses, most articles were rated as high quality (*n* = 27) while the remaining were rated as moderate. Analysis of codes (a presence of all components and factors in both high and moderate rated articles) demonstrated no differences between article quality rating. Further some moderate rated articles highlighted elements not observed in higher rated articles, confirming the decision to make no article exclusions.

### Quantitative synthesis

Most articles reported chronic illness research recruitment experiences related to cancer (*N *= 9), followed by older adults with multi-morbidities (n = 5) and cardiovascular conditions (*n* = 5). Other chronic illness represented included neurological conditions (*n* = 4), respiratory (*n* = 3), and other conditions (rheumatological *n* = 2, mixed pathologies *n* = 3, chronic pain *n* = 2, diabetes *n* = 2, and chronic rhinosinusitis *n* = 1). Summary descriptions were limited to participant type and recruitment screening outcomes (where reported/appropriate). A total of 2340 *people* reported on research recruitment experiences (see Table 3). There were five articles with multiple recruiting centres and reported factors affecting recruitment across those centres. For those studies, the sample number they reported was used in our summary, as this was the data available and grouped as “hospitals/clinics/nursing homes”.

**Table 3:** Description of participant types and reported recruitment data

Participant types	n	%
Clinicians	554	23.7%
Health care professional (not defined)	62	11.2%
Nurses	106	19.1%
Doctors	102	18.4%
Surgeons	57	10.3%
Other clinicians (physio, occupational, etc.)	2	0.4%
Hospitals\clinics\nursing homes	112	20.2%
Admin staff	17	3.1%
Managers	3	0.5%
Clinical setting declined original study	93	16.8%
Researcher	387	16.5%
Research nurses	130	33.6%
Researcher leads	49	12.7%
Number of trials#	34	8.8%
Pharma industry	25	6.5%
Other research team members	149	38.5%
Patients, carers, & representatives	1399	59.8%
Patients	972	69.5%
Participants who declined original study*	422	30.2%
Patient Representatives	2	0.1%
Carers	3	0.2%
Total	2340	

^#^Articles which looked at multiple studies recruitment

*Articles who collected information from participants who declined the original study but agreed to share their reasons for not participating

### Qualitative synthesis

**Fig. 3:**
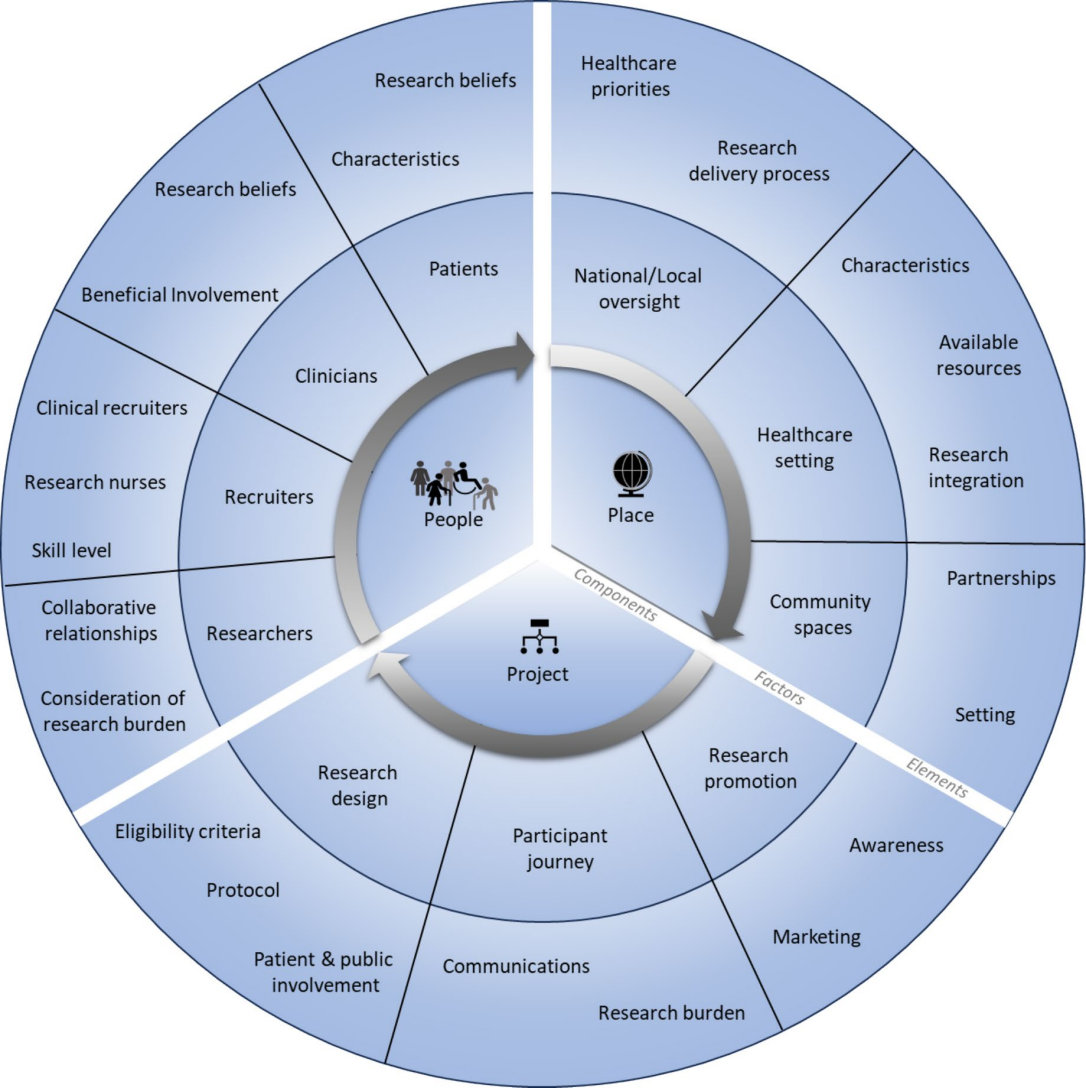
Taxonomy of research recruitment

The construction of the final taxonomy (Fig. 3) was formed through iterative evaluation of the final codebook and findings.

Identified as components to chronic illness research recruitment were defined:

1. People: all individuals involved in the design, delivery, and conduct of any research study.
2. Place: the environment where the research was conducted.
3. Project: the actual research project to which recruitment needs to occur.

The factors were defined as the subsections of each component. The factors in each component are:

1. People: patients, clinicians, recruiters, and researchers,
2. Place: national/local oversight, community, and healthcare settings,
3. Project: research promotion, design, and participant journey.

Within each factor, individual elements were identified which impacted research recruitment. Included articles typically reported impact as *facilitators or barriers* to research recruitment. Where this was observed, the impact was reported as originally described. The detailed elements are found in the outer ring in Fig. 3. Each component will be presented consecutively with factors and associated elements. An order for discussion was chosen, but these components are seen as a circular, where continual evaluation and adaptation may be required on a case-by-case basis.

### People

Four factors in this component are standard roles in research projects: 1) patients, 2) clinicians, 3) recruiters, and 4) researchers. Within the clinician and researcher roles as well as an independent role (e.g., research nurses or assistants, students, or administrators), a sub-role of recruiters was observed. The crossover between roles lead to the creation of the independent role of recruiters.

*Patients* were the first factor and included people (*n* = 1399) with a chronic illness. The elements observed to impact recruitment included 1) research beliefs and 2) individual characteristics.

1. Positive *research beliefs* were observed to facilitate recruitment for patients if they perceived benefits from research and held personal beliefs of the value of research. Benefits were either specific to the project (e.g., novel treatments, additional health screening, or home visits) [38, 39, 55, 56, 61] or generic to research participation (e.g., increased time with healthcare professionals, quality of care, and/or financial compensation) [38, 39, 55, 64]. The personal attribution of research value appeared related to their understanding of the research process, engagement with their health, and the belief that the research project met an unmet need [42, 47, 54, 55].
  *"In this disease, there is an unmet need for medical treatments. The patients are very much interested in participating in trials. (ID 24)” *[47]*.*
  If patients held negative beliefs around research, recruitment suffered. Negative research beliefs included concerns related to research participation (e.g., research intervention or processes worries [38–40, 43, 45, 46, 49, 51, 55, 58, 60, 65, 69], irrelevancy to the patient [38, 43, 47, 52, 54, 60, 65]) or generic non-interest in research (e.g., declining [38, 43, 44, 49, 54, 60, 65, 67, 71] and stated privacy worries [38, 49, 65]). Concerns related to research participation were observed as a barrier to recruitment in 13 articles. Concerns included randomisation to control/placebo arms [38, 43, 45, 46, 49, 51, 55, 65, 69], misunderstanding research processes [39, 40, 43, 55, 69], intervention treatment side effects or safety [43, 46, 49, 55, 60, 65], delivery of the intervention [38, 65], and cultural or historical perceptions of research [46, 55, 58]. Patient beliefs that research was irrelevant to them were typically related to their illness perception [38, 43, 54, 60, 65] or satisfaction with healthcare experiences [38, 47, 52].
  *“Participants expressed that they did not feel the study would be useful for them because they already had all the information they needed, already received the support they needed, had been on the same medications for a long time, or were on few medications.” Anzuoni, Field* [38].
  Patients non-interest in research was commonly reported as an expression of declining participation without a recorded reason or justification [38, 43, 44, 49, 54, 60, 65, 67, 71]. Privacy concerns of patients were related to medical and health data collection, use, and storage [38, 49, 65], as well as the authenticity of the invitation to participate [38] was reported to impact recruitment.
2. *Characteristics* of patients identified to impact on recruitment included their physical condition [38, 44–47, 49, 51, 60, 61, 65, 71, 73], current use of healthcare services [38, 44, 65, 71], carer consideration/permission [55, 65, 71], or available time [38, 44, 49, 61, 65, 69, 71]. Patients’ age [38, 60, 71], illness severity [38, 44–47, 49, 51, 60, 61, 65, 71, 73], literacy or education level [38, 71], and research beliefs (e.g., altruistic motivation) [38, 41, 49] were reported to alter willingness to be involved in research. Similarly, their current use of healthcare impacted willingness towards research participation related to their satisfaction with services or treatments [38, 65] or a trusted clinician’s approval regarding research participation [44, 65].
  Trust in research was observed to impact recruitment (*n* = 14 articles). If patients were worried about the reputation of research (i.e., fear of scams, historical injustice, fear of experimentation) [38, 39, 43, 48, 55, 60], the mode of data collection and storage [39, 43, 58] recruitment was negatively impacted. Trust could facilitate recruitment when the relationship between researchers, trusted clinicians and participants was strong [39, 42, 46–49, 51, 55, 56, 64], along with a professional project and staff reputation (e.g., uniforms, identification, webpage, consistent staffing) [39, 47, 51, 64].
  *“I think patient trust is the keyword for anything in this trial. Not even for this trial, for any trial. (Recruiter 6, research nurse)” *[49]*.*
  *Clinicians*, as the second factor, included multiple healthcare professionals (see Table 4). The elements identified to impact recruitment included 1) research beliefs and 2) beneficial involvement.

**Table 4:** Reported recruitment rate

Article	Number screened	Number consented	Recruitment rate (%)
Hall et al. [56]	384	128	33%
Whelan et al. [73]	281	26	9%
Hays et al. [57]	2565	22	1%
Lucas et al. [69]	949	75	8%
Duckham et al. [44]	3947	300	8%
Johnson et al. [68]	110	41	37%
McDermott et al. [49]	259	65	25%
Price et al. [70]	748	19	3%
Anzuoni et al. [38]	6470	361	6%
Edwards et al. [67]	128	19	15%
Tew et al. [72]	1968	299	15%
Brickey et al. [65]	11,029	483	4%
Stuckenschneider et al. [71]	1518	141	9%
Total	30,356	1979	7%

1. *Research beliefs* were observed to impact recruitment. A clinician’s research beliefs were informed by personal opinions of the research intervention [41–43, 45, 47, 50–55, 66, 68], research involvement motivation [43, 45–47, 50, 52, 54, 62, 67], opinions on patient participation [40, 41, 48, 50, 53, 62, 66, 67], and available time to perform research work [41, 43, 50, 52, 54, 71].
  Clinicians were reported to limit offering patients the opportunity to participate if the research project was viewed as having potential harm, uncertain outcomes, or limited future uptake in healthcare services [41, 43, 45, 47, 50–52, 54, 55, 66, 68]. Alternatively, if they held a favourable opinion of the research intervention [41, 42, 47] or as the only option for their patients [43, 47, 50–52, 54, 55, 66, 68] they would engage in recruitment.
  “Most [clinicians] cited that when there was a clear unmet need for the new drug treatment, trial subjects were found easily and recruitment was successful” [47].
  Clinician motivation to be involved in a research project also informed their research beliefs. If apathetic towards research, financial support was lacking, or high numbers of patients declined, motivation for recruitment waned [43, 47, 50, 62, 67]. In contrast, if clinicians were interested and dedicated to research recruitment efforts were consistent and often more successful [45–47, 50, 52, 54].
  Informing their research beliefs were clinical opinions on what participation would mean for patients. Clinicians were reported as utilising personal filters to eligibility criteria [40, 41, 50, 66, 67]. Personal filters could be as simple as age limitations despite the study having no upper limit or as complex as personal perception of the research interventions harm or benefit when the purpose of the study was to define outcomes. They were also observed to make assumptions around patients’ research participation across the multiple levels of involvement in research (discussed further in the factor of recruiters) [48, 50, 53, 66].
  *“Physicians having their own idiosyncratic inclusion criteria […] and that such physician strategies took recruitment out of recruiter’s hands” Brehaut, Lavin Venegas *[41]*.*
  Clinician’s personalised eligibility criteria included patient age [41, 54], ethnicity, culture, or language [40, 43, 48], illness severity [38, 40, 66, 67], mental health/competency [38, 40, 66], education, family support [40], geographic location [40], assumptions of patient suitability [40, 49, 50, 53, 66], and perceived ability to manage research requirements [40, 50, 53, 62].
2. *Beneficial involvement* was identified to impact recruitment for clinicians. When researchers collaborated with clinicians with trial design [50, 59] and in supporting research delivery [41, 50, 56] their engagement with recruitment was greater. Similarly, if clinicians felt that the research project improved clinical services [50, 54], increased staff education [50], or the department received financial compensation and/or token gifts [50, 52] recruitment activity was increased by clinicians.

*Recruiters* the third factor, were identified in both clinical and research teams. The recruiter roles were not consistently described between articles. While recruiters were identified as an independent factor, they bridged between the other roles on whom recruitment depends. Elements included 1) clinical recruiters, 2) research nurse/assistants, and 3) recruiter skill level.

1. C*linical recruiters* (e.g., doctors, nurses, administrative staff, etc.) were identified as clinically based staff who were actively involved in research recruitment processes. Their engagement in research recruitment activities was influenced by interdisciplinary involvement and support of the research study [48, 73], personal strategies to embed research into clinical practice [42, 49, 54, 56, 67], recruitment targets [54], patient responses to participation invitation [54], and diversity of clinicians approaching patients [64]. Different from the role in clinicians described in people, this element focuses on the active recruitment of patients to a study rather than the potential broader role of clinicians within a research project.
2. *Research nurses, or research assistants*, whose role is defined by research activities, were viewed as members of both clinical teams and research teams. Their engagement with research recruitment was reported to be influenced by staffing consistency and flexibility [38, 39, 41, 48, 49, 58, 70], research enthusiasm [39, 41, 45, 49], ability to build rapport [39, 41, 48], and ethnically inclusive attitude, approaches and skills [39, 48, 58, 64] impacted on recruitment. Alongside clinical recruiters, they reported how patient responses to research invites [41], recruitment targets [41], interdisciplinary involvement and support of the research study [41, 48–50, 70] altered their engagement with recruitment activities.
  Absence or presence of a research nurse [45, 47, 49, 50, 70, 72] was reported to impact recruitment. In particular, when research nurses were present and acted as advisors to clinical collaborators [40, 48–50], they were a key facilitating factor dependent on a clinician’s research skills and experience [49, 70]. On the contrary, absence of research nurses was reported to impact recruitment negatively.
  *“The [research nurse] has transformed our research […] she’s amazingly proactive and really made things a whole lot easier (Interview with Recruiter 5, surgeon)” *[49]*.*
3. In all recruiter roles, *skill level* impacted recruitment. Lower skill levels meant recruiters struggled with recruitment activities like approaching patients and study delivery tasks [41, 43, 45, 46, 48, 49, 54, 55]. Higher skill levels were observed together with greater confidence, involvement, and engagement with recruitment [39, 41, 45, 47, 50, 51, 54–56, 59, 64, 72]. There may be risks to recruitment in centres with more research experience due to competing studies [72].

*Researchers*, as the final factor, were described as those who were tasked to work on the study (*n* = 387). In this review this group included the researchers (e.g., academics, PhD candidates, etc.) leading the trials (n = 49 people), the wider research team (*n* = 149 people), clinical trials reporting on recruitment factors (n = 34 trials), industry partners (n = 25 partners), and sometimes research nurses (see recruiters). Key elements observed to influence recruitment included 1) collaborative partnerships and 2) consideration of research burden.

1. *Collaborative partnerships:* successful recruitment appeared to be built through relationships and research team leadership. Key for the formulation of collaborative partnerships was building relationships between clinicians and recruiters [41, 42, 47, 49, 60], researchers and recruiters [42, 47, 58, 62–64, 68], and patients with researchers, recruiters, and clinicians [47]. Relationships were developed on researchers demonstrating their commitment and approachability [42, 60, 62], rewarding clinical involvement (e.g., authorship, designing sub-study, token gifts) [43, 45, 59], co-working with clinical teams and other recruiting sites [47, 50, 58], strong relationships with key contact(s) [59, 68], and compliance with national and local research processes [59].
  Researcher team leadership appeared to impact building collaborative partnerships. Enthusiastic, genial leadership and communication were observed to facilitate recruitment [45, 50]. But weak, poor, and unclear leadership could form a barrier to recruitment [43, 45]. The lack of a collaborative partnership was confusing to participants altering their willingness to participate in research:
  “Where the unique divisions (medical vs research) are not clear. Consequently, the goals and objectives of the many individuals […] When interactions lack clarity, the likelihood of research enrolment and participation will decline.” Magwood, Ellis [60].
  Once established, these relationships were maintained through communication, training, and supportive research delivery. Communications from researchers reported to impact recruitment included study newsletters, email updates and recruitment progress [41, 45, 47, 49, 50, 54, 56, 64], regular reminders/meetings about eligibility criteria [47, 52, 56, 60, 64], and face-to-face meetings [45, 49, 52]. The lack of timely and effective communication was reported as a recruitment barrier [39, 45, 47, 49–51, 59].
  Research training provided by the researcher reported to impact recruitment included protocol training, specific research tests and procedures [39, 41, 43, 45, 48–50, 64, 68], research training for clinical leaders [39, 49, 64], cultural training [48, 56], and recruitment/consent training [41, 48–50].
  Supportive research delivery was identified as including: researcher oversight and support [39, 41, 45, 50, 54, 64], supportive study setup/opening [39, 50], timely and comprehensive renumeration related to study activities [39, 50], equipment/technical support [39, 50], and research mentorship [50].
2. *Consideration of research burden* was the second factor to impact recruitment for researchers. Research burden can affect both clinicians and participants when they consider engaging or participating with a research project. Activities reported to contribute to or alleviate the research burden were financial reimbursement for participants [61, 68] and staff [61]; combining research visits with existing clinic visits [47, 60, 61, 64, 68]; reducing research work requested of clinical collaborators [47, 61, 62] or participants [47, 61, 63, 64, 68]; and timing of research activities [55, 59, 61].

### Place

In this component, three factors were identified to impact recruitment related to the environments where the research was to be conducted: 1) national/local oversight, 2) healthcare setting, and 3) community spaces.

*National and/or Local Oversight* institutions are high-level bodies which often establish policies and processes around research. Their oversight was seen to impact recruitment by the following elements 1) research delivery process and 2) healthcare priorities.

1. *Research delivery processes* impacted recruitment through national research regulations/legislation [45–47], institutional ability to host research [40, 47], research approval processes [43, 45, 47, 58, 69], government funded research nurses [50] and competition between studies [41, 43, 45, 47, 58, 60] where commercial trials were given priority [41, 47].
2. *Healthcare priorities* were noted to impact recruitment by altering research culture through: high-level decision-making against research participation [55, 68], care pathway priorities [49, 54, 55, 68], prescribing or referral policies [49, 55], and perceived patient priorities [54].

*Healthcare settings*, as the second factor, were broadly represented in included articles (e.g., ward, clinic, hospital, nursing home, and GP surgeries). There were three key elements across all healthcare settings: 1) research integration, 2) available research resources, and 3) characteristics of the healthcare service.

1. *Research integration* was described as both within wider healthcare settings and as an individual clinician mindset (the latter being described previously in the component of people). Research integration impacted research referrals, alterations to workstreams, and screening behaviours [40, 41, 45, 47–50, 52, 54, 56, 64, 72]. Screening was described as searching through clinic lists or electronic health records, as well as creating clinical research repositories [47, 50, 52, 54, 56, 60, 69, 70]. Having a physical space in the clinical setting and streamlined integration of research team recruiters within clinical teams was observed to facilitate recruitment [41, 47, 49, 50, 56, 58, 59, 72].
  “Real-time clinic recruitment: The physical space of the clinic consisted of staff and faculty offices that were contiguous with the clinic rooms so that the patient could be recruited at the time of their clinical visit” Hall, Moore [56].
2. Lack of *available research resources* to perform research recruitment was the second element reported to impact recruitment. Resources were only discussed as a barrier to recruitment and included clinician research time or clinical pressures [38, 40, 43, 47, 50–54, 58, 60, 65, 67, 68, 70]; system capacity (e.g., space, equipment, finances, data management systems) [38, 40, 41, 43, 50, 51, 53, 58, 60, 68, 70]; and/or unavailability of a research nurse [43, 50, 58, 69, 70].
  “Time and work pressure were expressed by almost all non-, low-, and medium-recruiters and only sporadically by high-recruiters as barriers to recruitment efforts” Vluggen, Hoving [54].
3. *Characteristics of healthcare services* were the third element found to influence recruitment. Key characteristics noted included the presence or absence of the desired patient population [45, 47, 50, 60, 68–70], size of the service [60, 68, 72], and staffing [43, 50, 55].

*Community Spaces*: were observed as the third factor in Place. A community space was frequently noted as a facilitator for research recruitment, especially for ethnic minority populations. The elements described to impact recruitment were 1) settings and 2) partnerships.

1. Community *settings* were both physical (e.g., barbershops, bars, community support groups, illness associations or charities, churches, etc.) [39, 42, 44, 47, 57, 58, 61, 64] or virtual/remote settings (e.g., social media, letters, illness association websites/registers) [42, 44, 47, 57, 61].
2. Research projects that built *partnerships* with community groups observed how recruitment benefitted, particularly for individuals who may not regularly visit clinical spaces [47, 57]. Research project inclusivity and approachability were also improved [39, 58, 64]. Partnerships were established by building relationships [50, 56, 60, 62, 64] with key contacts [39, 42, 44, 47, 56–58, 60–62, 64].
  *“The study team treated the clinic staff as research partners and regularly communicated that the clinic staff members were valued as key to the research process. This enhanced the clinic staff engagement with the research process and, in turn, promoted their support of the recruitment and retention efforts.” Taani, Zabler *[64]*.*

### Project

The third component observed in this review was the project. Three factors related to this component were 1) design, 2) participant research journey, and 3) research promotional activities which appeared to impact recruitment.

*Design* of a research project impacts research recruitment through three elements: 1) eligibility criteria, 2) protocol characteristics, and 3) involvement of patients, public, and stakeholders.

1. *Eligibility criteria* were reported in 25 articles as impacting recruitment. Criteria that were too narrow/stringent [38, 43, 46, 47, 50, 52–54, 56, 57, 60, 64, 67, 68, 70] or if a participant pool was overestimated [43, 47, 50, 51, 54, 58, 60, 67] meant restricted recruitment. Altering the eligibility criteria was offered as a solution [64]. Related to the eligibility criteria is screening. Screening work is substantial (Table 4) [38, 44, 56–58, 60, 65, 67–70, 72, 73]. Not recognising screening work has important sequelae if the only recruiters are clinical collaborators without protected research time, as described in People and Place.
2. *Protocol characteristics* were reported as impacting on research recruitment. The complexity of a protocol [40, 41, 43, 45–47, 50, 54, 66, 68, 69] altered the willingness of both patients and clinicians to engage in recruitment. Unsupported logistics or procedures [39, 41, 43, 45–47, 50, 59] were observed to be barriers to recruitment, with clinicians experiencing frustration and embarrassment [50]. In contrast, if a protocol was compatible with clinical pathways [41, 43, 56] and offered flexibility for participants [41, 54, 63], this was reported to facilitate recruitment.
3. Only four articles reported how the *involvement of patients, the public, and key stakeholders* was related to recruitment. Involvement of patients and the public was reported as facilitating in both the design [42, 60, 68] and delivery of the trial [42, 47, 68]. Their involvement helped by creating culturally sensitive strategies [60] and ensuring alignment of research and patient priorities [42].

*Participant research journey*, as the second factor, refers to the pathway that participants take in research (e.g., number of visits, activities, etc.). Within the research journey, two elements were observed 1) communication and 2) research burden to impact research recruitment.

1. *Communications* were reported as key in building relationships with research participants [39, 42, 61, 70]. Effective communication was observed to impact recruitment through providing information on research progress and explaining outcomes [39–46, 49, 50, 52, 55, 64, 66]. The lack of communication of research results was reported to elicit negative feelings in participants [39, 42, 55]. In contrast, regular participant communications built strong relationships, reduced research burden, and imbued value [42, 55, 64]. Accessible patient-facing documents (information sheets and consent forms) were also reported to positively impact recruitment [40, 42, 46, 52, 55, 64, 67]. Here the role of patient representatives in improving documents was noted [42, 64].
  “Several oncologists believed the amount of information in consent forms was a hindrance to patients’ understanding and caused some patients to become “paralyzed,” unable to reach a decision” Bell, Kelly [40].
2. *Research burden* was reported as the participants’ perception of the work required of them due to research participation. Included articles multiple contributors to research burden were reported: time/convenience [39, 41, 43, 45, 46, 49, 50, 54–56, 58, 61, 69, 73]; lack of flexibility [39, 41, 43, 46, 50, 55, 56, 69]; task difficulty/complexity [42, 43, 50, 54–56, 58]; language barriers [45, 58]; financial costs (time off work, transport, etc.) [46, 49, 50, 55]; and/or risks and fears related to involvement [39, 42, 45–47, 49, 50, 58]. Higher levels of participant research burden could alter willingness to participate in research [42].

A protocol’s concordance with a patient clinical pathway was related to the research burden, where mismatches increased the research burden. Additionally, the timing of the research approach in a clinical journey altered willingness for patients to participate and clinicians to discuss research. A patient’s readiness for research appeared related to illness acuity or their hospital admission journey [38, 41, 43, 46, 49, 55, 60, 65]. Concurrently, clinician availability to discuss the research project impacted recruitment [40, 47, 50, 60, 70].

Incentives offered to patients for research participation were reported to offset research burden. Incentives included both monetary and non-monetary tokens (e.g., reporting of findings, gift or parking vouchers, additional health appointments, etc.) [39, 41, 42, 44, 45, 49, 50, 56–58].

*Research promotion* was the final factor in the Project component and appeared to increase research appreciation for collaborating clinicians and potential participants through promotional elements such as 1) marketing and 2) awareness.

1. The type of *marketing* activities ranged from national and community campaigns (e.g., television, radio, patient symposiums, etc.) [39, 42, 55–57] to posters in the recruitment setting [41, 54, 56]. Study logos were seen to raise awareness and increase research credibility [39]. While direct impact was not clear for a research project’s online presence, these activities were reported to increase research awareness [39, 42, 45, 61].
2. Higher levels of research *awareness* decreased the numbers of eligible patients who did not participate [43] and improved clinician referrals [50]. Regular meetings that focused on the justification for research and eligibility criteria enhanced research awareness in clinicians and subsequently impacted recruitment [49, 56].

### Taxonomy of recruitment

**Fig. 4:**
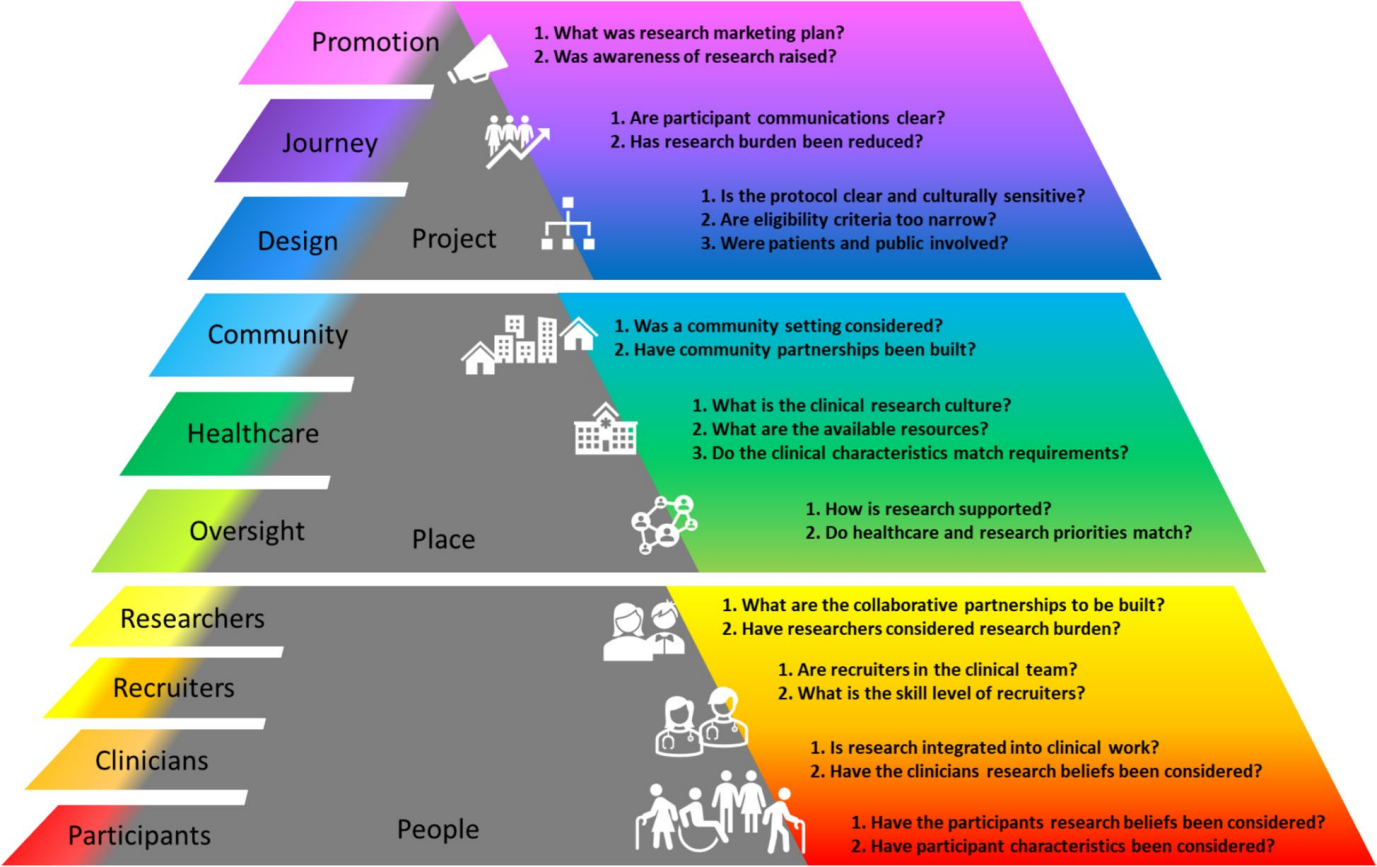
Practical questions to inform the design and reporting of recruitment strategies

Through an iterative process (RCA, BK, KHU) reached a consensus on the components, factors, and elements observed to impact research recruitment in chronic illness. The taxonomy (Fig. 3) was used to structure the practical questions for researchers to use to both inform and report recruitment strategies related to identified recruitment factors (Fig. 4). The practical questions were designed to encourage researchers to consider key concepts identified in this review and should be used in combination with the full taxonomy and codebook.

## Discussion

The restricted scoping review, by identifying and characterising the components, factors, and elements reported to impact research recruitment, created a chronic illness research recruitment taxonomy (CIRRT). The CIRRT was built from a heterogeneous group of recent scientific articles, creating a comprehensive and inclusive taxonomy.

In each component, factors were identified with specific elements reported to impact recruitment. In the component of people, factors included researchers, clinicians, recruiters, and participants roles. Elements ranged between research beliefs and the building of collaborative relationships. In the component of place, the factors included national or local research oversight institutions, healthcare environments, and community spaces. Identified elements ranged between setting characteristics and research delivery processes. Finally, in the component of project, factors included research design, participant research journey, and research promotion. Identified elements across this factor ranged from eligibility criteria to communication. A practical list of questions was to aid researchers in informing the design and reporting of research recruitment strategies.

This is the first study to bring all these concepts together in a single comprehensive taxonomy of chronic illness research recruitment. While other research recruitment frameworks exist, they focus on a singular component within CIRRT [20–23]. Corroborating the findings in CIRRT, other literature reviews reported similar factors that impact research recruitment related to participant experience [12, 15], clinical collaborator experience [7, 12], recruiter experience [14], research integrated into clinical pathways [74], and research delivery & design [12, 14, 15, 75].

Articles in this restricted scoping review included the perspective of older adults and minority ethnic groups, reputed as hard to reach, increasing the inclusivity and applicability of the offered taxonomy of chronic illness research recruitment. Similar to Savard and Kilpatrick [16], we identified how participant characteristics (e.g., physical and cognitive abilities, resource constraints, risks to participation, language, and labels) altered their ability to find and access opportunities for research participation.

Limitations included those inherent to both scoping [27] and restricted reviews [28]. The results of this review are subject to random and selection bias related to the applied restrictions and preliminary nature of this review. Specific limitations include the restrictions in the search strategy, the lack of a full verification process in screening, data extraction, MMAT score review, and the lack of protocol registration. Additional steps taken in this review to reduce bias were the verification of a random sample of articles by a second reviewer (AMLH, MS, KHU), seeking additional data sources through hand-searching reference lists, and performing a critical assessment of all included articles. While the additional restriction of a nursing focus helped to identify studies with a specific nursing research focus or methods, this may also have excluded other relevant non-nursing research.

Despite the existence of tools such as QRI [24] and PRECIS-2 [26], challenges of research recruitment remain. The QRI tool is an interventional tool designed to be prospectively embedded in clinical trials but it requires substantial additional research work, which may not be suitable for all research studies. The PRECIS-2 is a tool designed for trialists that provides a scoring system to rate their design choice’s ability to produce the required outcome for a pragmatic trial. The CIRRT provides a summary of factors identified to impact chronic illness research recruitment and places them into a detailed taxonomy for researchers to consider and customise to the design or reporting of their study. Taxonomies are recognised as crucial in informing knowledge through the provision of a structured frameworks and have to standardised and expanded multiple classifications in health research [76, 77]. By taking a general approach, the CIRRT might be applicable to a wide variety of chronic illness research studies and may help to inform both the creation and reporting of robust recruitment strategies. There have been calls for improved reporting structures for recruitment strategies [9, 12]. While the development of CIRRT represents a first step in this process, there is a need for further validation work, and caution should be used in widespread use. The taxonomy is not being suggested as identifying causal factors; rather, it provides structure for researchers to consider in the development and reporting of research recruitment strategies.

## Conclusions

Recruitment to chronic illness research remains an ongoing challenge. Recruitment strategies are an additional detail to effective research design. The taxonomy created in this restricted scoping review of 36 articles around the facilitators and barriers to research recruitment builds on previous research. It offers both a taxonomy and practical questions to inform the design of inclusive and robust recruitment strategies. The CIRRT may provide researchers a guide for reporting on recruitment strategies used in chronic illness research.

## Supplementary Information

Supplementary Material 1.

## Data Availability

Search Strategy: https://www.cabidigitallibrary.org/doi/10.1079/searchRxiv.2024.00516 All data included is available from the previously published research articles. The data analysed during the current study are available from the corresponding author on reasonable request. The code book is provided in supplemental materials to illustrate the coding used in this review.
