# Supplementary material for: Taxonomy of chronic illness research recruitment: a restricted scoping review
Source: BMC Health Serv Res. 2025 Jul 29;25:986. doi: 10.1186/s12913-025-13115-8 (PMC12305917; doi:10.1186/s12913-025-13115-8)
Supplement: Supplementary file 1 — Supplementary Material 1. [file 12913_2025_13115_MOESM1_ESM.docx]

Supplemental Material: Code book

| Initial code book | | | | | | Final code book | | | | | |
| --- | --- | --- | --- | --- | --- | --- | --- | --- | --- | --- | --- |
| Component | | | | | | | | | | | |
| People | | Place | | Project | | People | | Place | | Project | |
| Factors | | | | | | | | | | | |
| Participants | | Infrastructure | | Design | | Participants | | Oversight | | Design | |
| Clinicians | | Clinical environment | | Patient research journey | | Clinicians | | Healthcare setting | | Research journey | |
| Researchers | | Community environment | | Promotion | | Recruiters | | Community spaces | | Promotion | |
|  |  |  |  |  |  | Researchers | |  |  |  |  |
| Elements/Activities | | | | | | | | | | | |
| Participants | Beliefs about research | Infrastructure | Available resources | Design | Protocol considerations | Participants | Research beliefs | Oversight | Healthcare priorities | Design | Protocol |
|  | Trust in researchers and clinicians |  | Strategic direction |  | Patient and public involvement |  | Characteristics |  | Research delivery process |  | Patient and public involvement |
|  |  |  |  |  |  |  | Trust |  |  |  |  |
|  | Altruistic nature of the individual | Clinical environment | Research delivery |  | Eligibility criteria |  | Altruism | Healthcare setting | Characteristics |  | Eligibility criteria |
|  |  |  |  | Research journey | Clear communications | Clinicians | Research beliefs |  |  | Research journey | Communications |
| Clinicians | Previous experience |  |  |  |  |  | Clinical role in research |  | Available resources |  |  |
|  |  |  | Supportive actions of research |  |  |  | Benefits |  |  |  | Research burden |
|  | Research beliefs |  |  |  | Participant research burden | Researchers | Collaborative relationships |  | Research integration |  |  |
|  |  |  |  |  |  |  |  |  |  |  | Incentives |
| Researchers | Build collaborative partnerships | Community Setting | Appropriate setting | Promotion | Research visibility and awareness |  | Consider research burden | Community spaces | Setting | Promotion | Awareness |
|  |  |  |  |  |  | Recruiters | Clinical referrals or recruiters |  |  |  |  |
|  | Consider the burden |  | Community partnerships |  | Promotional materials |  | Research nurses or assistants |  | Partnerships |  | Tools |
|  |  |  |  |  |  |  | Skill level |  |  |  |  |

Illustrative Coding

| Component: People (36 articles, 732 data extracts) | | |
| --- | --- | --- |
| Factors | Elements | Illustrative data extract |
| Clinician (23 articles, 182 data extracts) | Benefits | *Financial gain:*  “it’s provided additional revenue [...] for the surgery which has allowed us to employ more staff, it’s had a number of indirect benefits to the practice as well. (Recruiter 5 Practice Manager)” Prout, et al. (62) |
|  |  | *Improved services:*  “most high-recruiters labelled participation as fitting in their organization’s vision and mentioned that eHealth [research] could serve as a medium to support self-management initiatives.” Vluggen, et al. (71) |
|  |  | *Staff Education:*  “taking part in the study is advantageous for appraisals, ‘I’ve got revalidation coming up. It’ll be mentioned’ (Recruiter 7, general practitioner, high recruiter).” Prout, et al. (62) |
|  |  | *Token Gifts:*  “Food/treats provided by study team” Schmidt, et al. (64) |
|  | Research Beliefs | *Motivation for research:*  “When the investigators were motivated, had a passion for the research topic in question, and were committed to recruitment, they were able to enroll patients as planned, even if it was not always easy.” Laaksonen, et al. (55)  “Due to the level of confidence and trust built between the primary oncologist and the patients/their families, the clinical trial is best introduced and explained by the treating physician, which requires physician enthusiasm and commitment as well as open communication with the research team.” Keruakous, et al. (53)  “Not all healthcare professionals recorded details as requested.” Edwards, et al. (47)  “The responses from the DONs [director of nursing] were ‘we are unable to participate in research,’ ‘this is not the right time for a research study,’ and ‘I’m sorry, we are just not interested’.” Shropshire, et al. (65) |
|  |  | *Opinion of the intervention*  Harmful: “Concern that participation in the trial might harm the patient” Isaksson, et al. (51)  Unsure: “doubts regarding the additive value of participation” Johnson, et al. (52)  Valuable: “HCPs [healthcare professionals] supported the intervention concept and thought it was of value to patients.” Stafford, et al. (66)  Only treatment option: “when there was a clear unmet need for the new drug treatment, trial subjects were found easily and recruitment was successful.” Laaksonen, et al. (55) |
|  |  | *Time pressure:*  “I forgot about the study” Schmidt, et al. (64)  “Too much work: ‘it did sound very complex and labour intensive’ (Recruiter 7, General Practitioner).” Prout, et al. (62) |
|  |  | *Opinion on patient participation*  Assumptions about patient interest:  “Trials with complex study protocols required CT [clinical trials] personnel to limit recruitment to patients whom they believed would be able to comprehend study information.” (Bell)  “HCPs acknowledged that while the intervention was required as close to diagnosis as possible, it was not necessarily a high priority for patients who were already processing a lot of new information.” (Stafford)  Clinical interpretations of eligibility criteria:  “All nurses, but especially non-recruiters, described their overall patient population as not eligible to participate; patients were commonly characterized as being too old and not digitally oriented” Vluggen, et al. (71)  “Some oncologists and research nurses described instances where patients had been deemed eligible by the MDT [multi-disciplinary team] but were not approached because of how they presented emotionally at their appointment, and other instances where patients were approached with hesitancy, based on judgements about their ability to cope with trial participation decisions.” Conefrey, et al. (43) |
|  | Involvement | “Ensure practice staff (including non-clinical staff) are consulted on trial design and delivery before start of trial” Prout, et al. (62) |
| Participants (28 articles, 258 data extracts) | Altruism | “More general benefits that recruiters highlighted to encourage participation included patients having an increased sense of control over their condition and that they would be helping others.” Brehaut, et al. (41) |
|  | Characteristics | *Carer considerations:*  “It’s way more complex in the case of women, because… the husband gets involved, the mother-in-law gets involved, the father-in-law gets involved and then the whole family gets involved… for a married woman especially. IDI24 (HCW)” Wharton-Smith, et al. (72)  “less than 10% of the cases, caregivers or relatives decided against participation” Stuckenschneider, et al. (67) |
|  |  | *Healthcare service access:*  “Those with visiting nurse services were less likely to enroll (OR = 0.64; 95% CI = 0.48–0.85) than those without such services.” Anzuoni, et al. (38) |
|  |  | *Physical condition:*  “The most common reason to decline participation during telephone contact was being ‘too unwell’.” Stuckenschneider, et al. (67)  “237 (47.0%) refused due to barriers related to illness severity’.” Brickey, et al. (42) |
|  |  | *Time pressure:*  “Of these patients, 98 (19.4%) stated that they were too burdened by other  appointments or did not have enough time to participate.” Brickey, et al. (42) |
|  | Research beliefs | *Intervention concerns:*  “Patients were said to have fears about why they were being treated differently from others, being offered the trial when most receive treatment within standard health services.” Wharton-Smith, et al. (72)  “Cultural beliefs linked to the fear of receiving an investigational drug or a placebo are commonly encountered.” Keruakous, et al. (53)  “For others, there may be a perception that research has negative consequences. For example, one patient noted ‘a neighbor had participated in previous research and died so she wouldn’t be interested in participated in research’.” Magwood, et al. (58) |
|  |  | *Not interested in research:*  “Patients typically exhibited a lack of interest in research participation (79 [15.7%]).” Brickey, et al. (42)  “Fifty of the 98 participants (51%) provided no reason for non-participation in the trial despite being deemed eligible.” (Duckham) |
|  |  | *Relevancy:*  “older individuals more likely to state that they did not need the intervention due to having a visiting nurse (16% vs 8%) or having a caregiver or spouse who took care of their medications (15% vs 6%).” Anzuoni, et al. (38)  “Some are not at the point of “readiness to take on one more thing” particularly when the explanation of the research may not have clear “meaningfulness” to the stroke survivor or to their care partner or immediate support system.” Magwood, et al. (58) |
|  |  | *Privacy concerns:*  “However, some patients reported a fear of privacy breach or had negative feelings towards research (8 [1.6%]).” Brickey, et al. (42) |
|  |  | *Benefits:*  “Other reasons included the opportunity to voice their opinions, the beneﬁt of ‘company’ that a home visit would offer, and valuing research.” Anzuoni, et al. (38)  “most commonly described potential benefits of the CT [clinical trial], which was reported to support individuals’ decisions to engage with it, was the perceived superior quality of care with the CT [clinical trial] than the SoC [standard of care]” Wharton-Smith, et al. (72) |
|  |  | *Valuable option:*  " ‘In this disease, there is an unmet need for medical treatments. The patients are very much interested in participating in trials.’ (ID 24)” Laaksonen, et al. (55) |
|  | Trust | “Participants’ primary concern was reassurance of safety and security in participating in the biomedical studies “I saw the gentleman and did not know what he is about I was very uncomfortable” (Participant, FGD High SES).” Bailey, et al. (39)  “Black patients have lived through many negative experiences such as Jim Crow laws and Civil Rights era that cause a wall that CRNs [clinical research nurse] ‘feel like we’re trying to knock down or get through (CRN for nine years, African American)’” Legor, et al. (56)  “Ras [research assistants] reported that individualized close contact with patients helped them to trust the surgeon and team around them. Patients were then more likely to consider participation in the RCT [randomised control trial].” Realpe, et al. (63) |
| Recruiters (23 articles, 112 data extracts) | Clinical role in research | “CT’s [clinical trials] best interest or the patient's best interest too, but I also have to think about the CT [clinical trial] because that's what my job is supposed to be.” Bell, et al. (40)  “the assigned nurse study coordinator had a 25-year history working with patients with [chronic illness type] and had a successful history of recruiting patients with [chronic illness type]. She was frequently available after hours to talk to possible participants, would attend symposia and recruiting events, and was willing to contact participants using social media or texting.” Hall, et al. (48)  “The clinical research team were most often informed about patients by specialist respiratory physiotherapists and nurses (49.8%) whilst remaining patients were referred by physiotherapists (27%), nurses (19.9%), doctors (2.8%) and other (0.3%).” Whelan, et al. (73) |
|  | Research nurses | “potential participants were not willing to wait for the blood tests due to work or other commitments, and therefore the speed and efﬁciency of the research nurses, and the number of research nurses on site was a factor in recruitment numbers.” Imran, et al. (50)  “The role of research nurses emerged as essential to ensure smooth flow so that a multiplicity of tasks fitted together into a cohesive process.” McDermott, et al. (59) |
|  | Skill level | *Novice:*  “The magnitude of patients declining the invitation to participate evoked negative emotional and practical responses in most non- and low-recruiters and half of the medium-recruiters.” Vluggen, et al. (71)  “Clinicians lacking necessary research experience, skills or training (e.g. GCP [good clinical practice])” Crocker, et al. (45) |
|  |  | *Experienced:*  “Investigators’ experience of the investigational drug from earlier trials or a belief in the drug’s potential benefit for patients influenced the recruitment success.” Laaksonen, et al. (55)  “The practice sites identified with the 25% lowest recruitment cost (n = 7) were significantly more likely to be more experienced in research participation (100% vs. 44.44%).” Tew, et al. (70) |
| Researchers (22 articles, 180 data extracts) | Collaborative relationship | *Interactions with clinicians*  Training: “Almost all recruiters indicated they did not receive formal training, and several felt they would have benefitted from such training.” Brehaut, et al. (41)  “Participants discussed that cultural training received from their organizations was usually a yearly, on-line training that was described as generalized and outdated.” Legor, et al. (56)  Supportive research delivery: “In addition to this supervisory type of support, field staff also described support needs in terms of the study setup. They spoke about late remuneration, lack of transportation to carry study equipment during data collection, and periodical faulty equipment.” Bailey, et al. (39)  “Suggest to practices the possibility of engaging with a research practice mentor, one who has experience in participating in trials.” Prout, et al. (62)  Communication: “that the support team responds quickly to questions (89%)” Isaksson, et al. (51)  “Mutual encouragement and good team communication were described by most recruiters as important in maintaining motivation and effective teamwork.” McDermott, et al. (59) |
|  |  | *Relationship building*  “Building relationships with local urology teams could help to stimulate interest and recruitment” Sullivan, et al. (68)  “research nurses faced challenges in terms of a lack of support by local teams. However, overtime working alongside [chronic illness type] clinic nurses and staff, acceptance and co-working was established by sharing workloads.” Imran, et al. (50) |
|  |  | *Research team leadership*  “Poor central management/oversight of trial” Crocker, et al. (45)  “Genial nature of research team: helpful we’ve had a lot of support from the team.” Prout, et al. (62) |
|  | Consideration of research burden | “Fewer patients are seen in hospitals during these festivals, so these may not be ideal times to start collecting data.” Koirala, et al. (54)  “To help mitigate patients from being overburdened with research participation request, database information fields should include dates of contact and enrolment status (declined, pending, or active).” Magwood, et al. (58) |
| Component: Place (34 articles, 266 data extracts) | | |
| Factors | Elements | Illustrative data extract |
| Community spaces (13 articles, 61 data extracts) | Partnerships | *Collaborations*  “Each community physician also received a letter from the study team when the individual enrolled, thanking them for the referral and detailing the specifics of the study.” Hall, et al. (48)  “The study team treated the clinic staff as research partners and regularly communicated that the clinic staff members were valued as key to the research process. This enhanced the clinic staff engagement with the research process and, in turn, promoted their support of the recruitment and retention efforts.” Taani, et al. (69) |
|  |  | *Key contacts*  “Village managers within all three cohorts did not provide permission for the recruitment of participants to be extended to non-village residents that fulfilled the inclusion criteria” Duckham, et al. (46)  “Project champions are individuals who are well known and connected within the community and committed to the success of the proposed study. These individuals actively recruit participants, announce project events, and sustain interest and participation throughout the study.” Nichols, et al. (60) |
|  | Setting | “Since we knew that this age group responds better to technology-based recruitment, we switched  from mailing research packets to an online format and used SurveyMonkey.” Hays, et al. (49)  “only their in-person recruitment efforts yielded enrolment of study participants. The free clinic patients did not respond to either the study outreach flyers or free clinic provider referrals to the study.” Taani, et al. (69) |
| Healthcare setting (26 articles, 161 data extracts) | Available resources | “current pressures on the National Health Service (NHS), meaning that clinical staff do not have time or resources to give to trial recruitment” Crocker, et al. (45)  “A GP [general practitioner] from a ‘high’ recruiting practice said that they had had to increase the hours of one of their practice Nurses and also take on a locum nurse.” Prout, et al. (62) |
|  | Characteristics | “There were not enough patients coming through the department with marker conditions during the time we were there. I couldn’t find one patient on Saturday afternoon.” Price, et al. (61) |
|  | Research integration | “Real-time clinic recruitment: The physical space of the clinic consisted of staff and faculty offices that were contiguous with the clinic rooms so that the patient could be recruited at the time of their clinical visit.” Hall, et al. (48) |
| Oversight (14 articles, 44 data extracts) | Healthcare priorities | “Tension may emerge within systems where two dis-tinct practices coexist, for example, according to the CT [clinical trial] guidelines, patients could be hospitalised for 14 days however, the local regulations or “prikaz” could supersede this and force the patient to stay longer.” Wharton-Smith, et al. (72)  “It’s been initiated in some other practices, [apps and such things] but well, it’s just neither a relevant nor a urgent issue here” Vluggen, et al. (71) |
|  | Research delivery process | “Another barrier mentioned by one recruiter involved recruiting for multiple competing studies, leading to a conflict” Brehaut, et al. (41)  “Delays in local R&D/Trust approvals” Crocker, et al. (45) |
| Component: Project (36 articles, 401 data extracts) | | |
| Factors | Elements | Illustrative data extract |
| Participant research journey (30 articles, 264 data extracts) | Communications | “the recruiter uses simple direct language as recommended by the recruiter training to communicate equipoise (‘we don’t know which one is best’) and to explain randomisation (‘equal chance of getting them’).” McDermott, et al. (59)  “Consent forms were also described as problematic, causing CT personnel to decide who was most capable of comprehending the forms prior to offering and discussing a trial” Bell, et al. (40) |
|  | Incentives | “This included on-site information sessions, an offer of a free 30-min muscle health and functional assessment, and individually addressed study information packs mailed to the residents via the village managers.” (Duckham)  “Qualifying participants had the option to provide their mailing addresses after completion of the surveys to receive a $20 Amazon gift card to reimburse their time.” Hays, et al. (49) |
|  | Pathway integration | “those who declined the initial invitation was timing of the invitation relative to leaving the hospital; focus group participants indicated that if the request had come later, they may have been more likely to say yes (N = 3).” Anzuoni, et al. (38) |
|  | Research burden | “The length of the questionnaire and corresponding time needed to complete was identified as a challenge to participation.” Bailey, et al. (39)  “nine of whom withdrew, because the times offered for the intervention were inconvenient” Lucas, et al. (57) |
| Research design (33 articles, 113 data extracts) | Eligibility criteria | “narrowly defined criteria for inclusion and exclusion” Isaksson, et al. (51)  “Comorbidities is often what excludes patients from participating in clinical trials” Keruakous, et al. (53) |
|  | Patient and public involvement | “Our patient partners were also really critical in the design of the recruitment materials.” Coyle, et al. (44)  “As a pragmatic, patient-centered trial, [name of trial] incorporated patient perspectives throughout the study design, including an iterative consenting design process” Johnson, et al. (52) |
|  | Protocol | “Complex vs simple trial design was viewed by CT [clinical trial] personnel to impact patients’ ability to understand what was involved in CT participation.” Bell, et al. (40)  “The problem with [name of trial] and some of these studies is that you have to consent now. You do not, you know, we cannot push it back a week.” Brehaut, et al. (41)  “Study protocol incompatible with clinical practice/hospital policies” Crocker, et al. (45) |
| Trial promotion  (9 articles,  24 data extracts) | Awareness | “Raising awareness of [name of trial] trial through presentations to GP meetings and/or informal networking was found to be successful in some areas.” McDermott, et al. (59)  “Eligible patients not aware of opportunity to take part (e.g. due to poor advertising or clinician gate-keeping” Crocker, et al. (45) |
|  | Marketing tools | “flyers throughout the community, in such places as restaurants, library, and church bulletin boards, announcements at the senior center or congregate living site, and articles in local newspapers” Nichols, et al. (60) |
